# Porcine sapovirus Cowden strain enters LLC-PK cells via clathrin- and cholesterol-dependent endocytosis with the requirement of dynamin II

**DOI:** 10.1186/s13567-018-0584-0

**Published:** 2018-09-17

**Authors:** Mahmoud Soliman, Deok-Song Kim, Chonsaeng Kim, Ja-Young Seo, Ji-Yun Kim, Jun-Gyu Park, Mia Madel Alfajaro, Yeong-Bin Baek, Eun-Hyo Cho, Sang-Ik Park, Mun-Il Kang, Kyeong-Ok Chang, Ian Goodfellow, Kyoung-Oh Cho

**Affiliations:** 1 Laboratory of Veterinary Pathology, College of Veterinary Medicine, Chonnam National University, Gwangju, Republic of Korea; 2 Korea Research Institute of Chemical Technology, Daejeon, Republic of Korea; 3 Department of Diagnostic Medicine and Pathobiology, College of Veterinary Medicine, Kansas State University, Manhattan, KS USA; 4 Division of Virology, Department of Pathology, University of Cambridge, Cambridge, UK

## Abstract

Caliciviruses in the genus *Sapovirus* are a significant cause of viral gastroenteritis in humans and animals. However, the mechanism of their entry into cells is not well characterized. Here, we determined the entry mechanism of porcine sapovirus (PSaV) strain Cowden into permissive LLC-PK cells. The inhibition of clathrin-mediated endocytosis using chlorpromazine, siRNAs, and a dominant negative (DN) mutant blocked entry and infection of PSaV Cowden strain, confirming a role for clathrin-mediated internalization. Entry and infection were also inhibited by the cholesterol-sequestering drug methyl-β-cyclodextrin and was restored by the addition of soluble cholesterol, indicating that cholesterol also contributes to entry and infection of this strain. Furthermore, the inhibition of dynamin GTPase activity by dynasore, siRNA depletion of dynamin II, or overexpression of a DN mutant of dynamin II reduced the entry and infection, suggesting that dynamin mediates the fission and detachment of clathrin- and cholesterol-pits for entry of this strain. In contrast, the inhibition of caveolae-mediated endocytosis using nystatin, siRNAs, or a DN mutant had no inhibitory effect on entry and infection of this strain. It was further determined that cell entry of PSaV Cowden strain required actin rearrangements for vesicle internalization, endosomal trafficking from early to late endosomes through microtubules, and late endosomal acidification for uncoating. We conclude that PSaV strain Cowden is internalized into LLC-PK cells by clathrin- and cholesterol-mediated endocytosis that requires dynamin II and actin rearrangement, and that the uncoating occurs in the acidified late endosomes after trafficking from the early endosomes through microtubules.

**Electronic supplementary material:**

The online version of this article (10.1186/s13567-018-0584-0) contains supplementary material, which is available to authorized users.

## Introduction

Viruses are obligatory intracellular parasites, and so must deliver their genetic material into host cells to initiate infection. This requires the specific recognition of cell surface molecules and the subsequent entry process [1]. The mechanisms by which viruses gain entry into host cells are diverse and include direct penetration through the plasma membrane or endocytic uptake followed by vesicular transport through the cytoplasm and delivery to endosomes and other intracellular organelles [2]. The internalization process can occur via clathrin-mediated endocytosis, caveolar/lipid raft-mediated endocytosis, macropinocytosis, or a variety of other still poorly characterized mechanisms [2].

Clathrin-mediated endocytosis is generally accepted to be a major route by which nonenveloped viruses infect cells [2]. This route is utilized by a variety of viruses, such as adenovirus [3], canine parvovirus [4], and picornaviruses including rhinovirus [5] and foot-and-mouth disease virus [6]. In many cases and even within the same family, viruses utilize different endocytic pathways. As examples, parechovirus 1 and coxsackievirus B3 (CVB3) use caveolae to infect cells [7, 8], while human echovirus 11 and CVB4 enter cells in a cholesterol-dependent manner [9, 10]. Furthermore, adeno-associated virus type 5, Japanese encephalitis virus, and influenza virus use more than one entry mechanism [11–13].

Caliciviruses are small, nonenveloped viruses of 27–35 nm in diameter, which enclose a single-stranded, positive sense RNA of 7–8 kb [14]. Caliciviruses have been formally classified into five genera: *Norovirus*, *Sapovirus*, *Vesivirus*, *Lagovirus*, and *Nebovirus* [14]. In addition, six new genera named *Recovirus* [15], *Bavovirus* [16, 17], *Nacovirus* [17–19], *Salovirus* [20], *Sanovirus* [21], and *Valovirus* [22] have been proposed. Sapoviruses (SaVs) and noroviruses are important etiologic agents of both human and animal viral gastroenteritis. Due to low propensity of enteric caliciviruses to grow in vitro, there is still limited understanding of their biology. Porcine sapovirus (PSaV) Cowden strain, a genogroup III SaV, is the only strain within the *Sapovirus* genus that replicates efficiently in vitro [23].

Caliciviruses vary in their mechanisms used for cell entry and replication. For example, feline calicivirus (FCV) uses clathrin-mediated endocytosis for cell entry [24], whereas murine norovirus-1 (MNV-1) uses dynamin and cholesterol-linked pathways [25, 26]. Following entry, the events that facilitate release of the calicivirus positive-sense RNA genome into the cytoplasm remain unclear. FCV replication is sensitive to chloroquine-mediated inhibition of vesicular-acidification, suggesting that release of the viral RNA into the cytoplasm requires a low pH environment [24]. In addition, FCV binding to the cellular receptor JAM-1 induces a conformational change in the capsid, indicating that uncoating may, in part, be receptor-binding mediated [27]. In contrast, MNV-1 infectivity does not depend on the acidification of endosomes [25, 28].

We have recently demonstrated that bile acids, endosomal acidification, and cathepsin L activity are required for PSaV uncoating and genome release into the cytoplasm [29, 30]. However, the detailed mechanism of PSaV entry into the host cells is yet to be determined. In the current study, we examined the entry process of PSaV using the cultivable Cowden strain as a model system. We demonstrate that the PSaV Cowden strain enters the permissive porcine kidney LLC-PK cells via clathrin- and cholesterol-dependent endocytic pathways, in a process that requires both dynamin II and actin for vesicle internalization. We further demonstrate that PSaV Cowden strain requires the Ras-related protein 5 (Rab5) and Rab7 GTPases to deliver virus cargo from the early endosomes (EEs) to the late endosomes (LEs) via microtubules, and that late endosomal acidification is essential for virus uncoating. These findings represent new insights into PSaV Cowden strain cell entry process.

## Materials and methods

### Cells and viruses

Porcine kidney LLC-PK and human cervical cancer HeLa cells obtained from the American Type Culture Collection (ATCC, Manassas, VA, USA) were maintained in Eagle’s minimal essential medium (EMEM). Human intestinal Caco-2 cells (ATCC) were grown in Dulbecco’s modified Eagle’s medium (DMEM). Monkey kidney MA104 cells (ATCC) were grown in alpha minimal essential medium (α-MEM). Each culture medium was supplemented with 10% fetal bovine serum (FBS), 100 U/mL penicillin, and 100 µg/mL streptomycin. The PSaV Cowden strain was generated from the full-length infectious clone pCV4A and was propagated in LLC-PK cells in the presence of 200 μM GCDCA [23]. CVB3 Nancy strain (ATCC) was propagated in HeLa cells [31] and then used for the subsequent studies with Caco-2 cells [31]. The human rotavirus strain Wa (G1P [8]) (ATCC) was preactivated with 10 μg/mL crystalized trypsin and propagated in MA104 cells as previously described [32].

### Reagents and antibodies

Chlorpromazine, chloroquine, and GCDCA purchased from Sigma-Aldrich (St. Louis, MO, USA) were dissolved in distilled water (DDW). Nystatin, methyl-beta-cyclodextrin (MβCD), cytochalasin D, nocodazole, and amiloride (Sigma-Aldrich) were dissolved in dimethyl sulfoxide (DMSO). Alexa Fluor 594 (AF594) succinimidyl ester purchased from Molecular Probes (Eugene, OR, USA) was dissolved in DMSO. AF488-labeled phalloidin, 5-chloromethylfluorescein diacetate (CMFDA) pH probe, and slowFade Gold antifade reagent with 4′,6-diamidino-2-phenylindole (DAPI) were obtained from Molecular Probes (Eugene, OR, USA). Mouse monoclonal antibodies (Mabs) against each clathrin heavy chain, caveolin-1, and early endosome antigen 1 (EEA1) were purchased from BD Transduction Laboratories (Lexington, KY, USA). Rabbit anti-dynamin 2 and rabbit anti-Rab7 polyclonal antibodies, and mouse anti- lysosome-associated membrane protein-2 (LAMP2) Mab were purchased from Abcam (Cambridge, MA, USA). Rabbit anti-Rab5 polyclonal antibody was obtained from Cell Signaling Technology (Beverly, MA, USA). Rabbit anti-glyceraldehyde 3-phosphate dehydrogenase (GAPDH, FL-335) polyclonal antibody was from Santa Cruz Biotechnology (Dallas, TX, USA). Rabbit anti-PSaV VPg polyclonal antibody [33], mouse anti-CVB3 capsid Mab (Millipore, Bedford, MA, USA), and mouse anti-rotavirus VP6 Mab (Median Diagnostic, Chuncheon, South Korea) were used to detect the corresponding viruses. Secondary antibodies included horseradish peroxidase (HRP)-conjugated goat anti-rabbit IgG (Cell Signaling Technology), HRP-conjugated goat anti-mouse IgG (Ab Frontier, Seoul, South Korea), fluorescein isothiocyanate (FITC)-conjugated anti-rabbit IgG, and FITC-conjugated anti-mouse IgG antibodies (Santa Cruz Biotechnology) were used.

### Cytotoxicity assay

The cytotoxicity of the chemicals used in this study was determined using the 3-(4,5-dimethylthiazol-2-yl)-2,5-diphenyl tetrazolium bromide (MTT) assay as described elsewhere [34]. Briefly, confluent LLC-PK, Caco-2, and MA104 cells grown in 96 wells were incubated with medium containing different concentrations of inhibitors for 24 h. After removal of the media, 200 μL of MTT solution was transferred to each well and incubated for 4 h at 37 °C in a CO_2_ incubator. Afterward, 150 μL DMSO was added to each well and incubated at room temperature for 10 min. The absorbance was read in an ELISA reader with the optical density (OD) value of 570 nm. The percentage of cell viability was calculated as: [OD_(sample)_ − OD_(blank)_/(OD_(control)_ − OD_(blank)_] × 100. Non-toxic concentrations of each chemical were used in this study.

### Labeling of PSaV with AF594 and virus quantitation by transmission electron microscopy (TEM)

AF594 labeling of PSaV particles purified by cesium chloride (CsCl) density-gradient centrifugation was performed as described elsewhere [34]. Briefly, purified PSaV particles (10 mg at 1 mg/mL) in 0.1 M sodium bicarbonate buffer (pH 8.3) was mixed with one tenth fold-molar concentration of AF594 succinimidyl ester (1 mg at 1 mg/mL in DMSO) by vortexing for 30 s. The mixture was incubated for 1 h at room temperature with continuous stirring. Labeled PSaV particles were repurified with CsCl density-gradient centrifugation, dialyzed against virion buffer, and stored in aliquots of 2 µg at −20 °C. The AF594-labeled virus particles were counted along with the latex beads (Sigma-Aldrich) in at least 10 randomly chosen squares on the grid. The total virus count was calculated by the ratio of the virus particle number to the latex particle number and multiplied by the known latex particle concentration per mL.

### Treatment of cells with chemical inhibitors

Cells grown in 12-well plates or 8-well chamber slides to the desired confluency were washed twice with phosphate-buffered saline (PBS, pH 7.2). Subsequently, the cells were pretreated with various non-toxic working concentrations of the chemicals (Table 1) for 1 h at 37 °C. After washing the cells twice with PBS, the cells were infected with AF594-labeled or mock-labeled virus for the indicated times. Internalization, infectivity, NR infectious center assays were carried out as described below. Mock and control treatments were performed simultaneously.

**Table 1:** **Effects and dosages of chemicals used in this study**

Chemicals	Effects	Dosages			Solvent
		LLC-PK cells	Caco-2 cells	MA104 cells	
Chlorpromazine	Inhibits clathrin-mediated endocytosis	1 μM, 10 μM, 20 μM	1 μM, 10 μM, 20 μM	1 μM, 10 μM, 20 μM	DW
Methyl-ß-cyclodextrin	Depletes cholesterol	1 mM, 10 mM, 20 mM	0.5 mM, 1 mM, 5 mM	1 mM, 5 mM, 10 mM	DW
Soluble cholesterol	Replenish cholesterol	10 μM, 100 μM, 200 μM	10 μM, 50 μM, 100 μM	10 μM, 50 μM, 100 μM	DW
Nystatin	Inhibits caveolae-mediated endocytosis, sequesters cholesterol	1 μM, 10 μM, 25 μM	1 μM, 10 μM, 25 μM	1 μM, 25 μM, 50 μM	DMSO
Dynasore	Inhibits dynamin	1 μM, 50 μM, 100 μM	1 μM, 50 μM, 100 μM	1 μM, 50 μM, 100 μM	DMSO
Chloroquine	Inhibits acidification of endosomes	1 μM, 50 μM, 100 μM	–	–	DW
Cytochalasin D	Disrupts actin cytoskeleton	0.5 μM, 1 μM, 5 μM	–	–	DMSO
Nocodazole	Disrupts microtubules	1 μM, 10 μM, 20 μM	–	–	DMSO
Amiloride	Inhibits macropinocytosis	1 μM, 50 μM, 100 μM	–	–	DMSO

DW, distilled water; DMSO, dimethyl sulfoxide.

### Small interfering (si)RNAs and plasmid transfection

siRNAs targeting clathrin heavy chain, dynamin II, caveolin-1, Rab5, and Rab7, or scrambled siRNA were purchased from Santa Cruz Biotechnology (Additional file 1) [35–40]. Rab5 and Rab7 siRNAs were used at 20 nM concentration. Other siRNAs were used at 100 nM. Plasmid constructs of green fluorescent protein (GFP)-tagged-wild type (WT) and DN Eps15 were kindly provided by Alice Dautry-Vautry (Pasteur Institute, Paris, France) [41]. GFP-tagged WT and K44A DN dynamin II were kindly provided by Mark McNiven (Mayo Institute, Rochester, MS, USA) [42]. GFP-tagged WT and DN caveolin were kindly provided by Ari Helenius (Swiss Federal Institute of Technology, Zurich) [43]. Transfection of siRNAs or plasmids was performed using Lipofectamine 2000 (Invitrogen, Carlsbad, CA, USA) according to the manufacturer’s instructions [30]. To ensure effective siRNA knockdown of each target protein, transfected cells harvested at 24 h or 48 h post-transfection were analyzed by Western blotting analysis.

### Virus internalization assay

Mock-, chemical-treated, or siRNAs-transfected LLC-PK cells in 8-well chamber slides were inoculated with AF594-labeled PSaV Cowden strain (approximately 415 particles per cell) for 30 min at 4 °C. The cells were then shifted to 37 °C for the indicated times to allow virus entry to proceed. The cells were then fixed with 4% paraformaldehyde in PBS for 15 min at 20 °C and incubated with AF488-labeled phalloidin (10 units) for 15 min at 20 °C for cytoskeleton staining. For colocalization with endosomal markers, the cells were then permeabilized by the addition of 0.2% Triton X-100 in PBS for 10 min at 20 °C and washed with PBS containing 0.1% new-born calf serum (PBS-NCS). The cells were then incubated with Mabs against EEA1 and LAMP2 (1:100 dilution) at 4 °C overnight, washed twice with PBS-NCS, and incubated with FITC-conjugated anti-mouse IgG antibody (1:100 dilution) for 1 h at room temperature. The cells were mounted with SlowFade Gold antifade reagent containing 1 × DAPI solution for nucleus staining. Infected cells were observed with a LSM 510 confocal microscope and analyzed using LSM software (Carl Zeiss, Jena, Germany).

The low-pH rescue experiment was performed as described previously [44]. Briefly, mock- or chloroquine-treated LLC-PK cells infected with AF594-labeled PSaV particles (approximately 415 particles per cell) were incubated with either neutral (pH 7.2) or acidic (pH 5) citrate buffers for 5 min at 37 °C. After washing three times with PBS, the cells were incubated with EMEM with 2.5% FBS and 100 μM GCDCA for 2 h at 37 °C. Subsequently, the infected cells were fixed, permeabilized and stained with anti-LAMP2 antibody as described above.

### Detection of acidic intracellular compartments

The cell tracker green CMFDA pH probe was used to visualize intracellular acidic compartments as described elsewhere [40, 45]. Briefly, LLC-PK cells treated with or without chloroquine or infected with or without PSaV Cowden strain were washed with PBS and were incubated with CMFDA working solution (10 μM) for 30 min at 37 °C, which was then replaced with maintenance medium and incubated for another 30 min at 37 °C. Fixation and permeabilization were then performed as described above. To check the colocalization with late endosome marker, the cells were incubated with anti-LAMP2 antibody and examined by confocal microscope as described above.

### Immunofluorescence assay for the determination of virus infectivity

Infectivity assays were carried out as previously described [46]. Briefly, confluent monolayers of cells in 8-well chamber slides, which were not pretreated or pretreated with chemicals, or which were transfected with or without siRNAs, WT, or DN plasmids, were uninfected or infected with each virus. Virus inocula were then removed and cells were washed twice with PBS. Cells were incubated with PSaV Cowden strain for 24 or 36 h, with CVB3 Nancy strain for 4 h or 6 h, or with rotavirus Wa strain for 8 h or 15 h. The cells were then fixed with 4% paraformaldehyde in PBS for 15 min at room temperature, permeabilized by the addition of 0.2% Triton X–100 for 10 min at room temperature, and washed three times with PBS containing 0.1% PBS-NCS. The chamber slides were then incubated with primary antibodies (anti-PSaV VPg, anti-CVB3 capsid, or anti-RVA VP6) at 4 °C overnight. Subsequently, cells were washed three times with PBS, and FITC-conjugated secondary antibodies were added. After washing with PBS-NCS, the nuclei were stained with DAPI, and cells were examined using confocal microscopy. The number of infected cells was checked in 500 cells [26]. After image analysis with Zeiss LSM image browser (Oberkochen, Germany), the infected cells were counted as positive for viral antigen if they had a fluorescent intensity at least three times that of the uninfected controls. The percentage of positive cells was then normalized to that of the untreated control.

### Neutral red (NR) infectious center assay (uncoating assay)

NR-labeled PSaV particles were produced as described elsewhere [31, 47]. Briefly, LLC-PK cells were infected with the PSaV Cowden strain [multiplicity of infection (MOI) of 1 focus forming units (FFU)/cell] in 10 μg/mL NR. After incubation for 72 h, NR-labeled PSaV were harvested and purified in the dark. The NR infectious center assay was performed as previously described [31, 47]. Briefly, confluent monolayers of LLC-PK cells grown in 6-well plates were pretreated with chemicals or transfected with siRNAs. The cells were then infected with NR-PSaV for the indicated times at 37 °C in the dark and then exposed to ultraviolet (UV) light at 120 mJ/cm^2^ at 20 °C. Duplicate monolayers were maintained in the dark and used as unilluminated controls. The cells were detached with trypsin–EDTA, and the dissociated cells were counted, diluted, and replated onto fresh LLC-PK monolayers in 6-well plates. Each well was overlaid with agarose at 3 h post-infection. After 4 days, plaques that had developed were counted. In each of three independent experiments, the numbers of plaques in the chemical-treated or illuminated wells were normalized to the number of plaques in the unilluminated control (expressed as a percentage).

### Preparation of cell extracts and Western blotting analysis

Confluent LLC-PK cells in 6-well plates infected with or without PSaV Cowden strain, or transfected with or without siRNAs were harvested. The cells were washed twice with PBS and lysed with a cell extraction buffer (Invitrogen) supplemented with protease and phosphatase inhibitors (Roche, Basel, Switzerland). After centrifugation at 12 000 × *g* for 10 min at 4 °C, the supernatants of the cell lysates were normalized for equal protein content using a BCA protein assay kit (Thermo Scientific, Waltham, MA, USA). Total cell lysates were denatured and resolved by sodium dodecyl sulfate–polyacrylamide gel electrophoresis (SDS-PAGE). The resolved proteins were transferred to nitrocellulose blotting membranes (Amersham Protran; GE Healthcare Life Science, Little Chalfont, UK) and immunoblotted with primary antibodies specific for each target protein as described above. After incubation with secondary antibodies against mouse or rabbit IgG, immunoreactive bands were developed using an enhanced chemiluminescence reaction kit (DoGen, Seoul, South Korea), and images were obtained using the Davinch–Western imaging system (Young Ltd., Seoul, South Korea).

### Virus titration using median tissue culture infective dose (TCID_50_) assay

LLC-PK cells in 12-well plates transfected with or without siRNAs were infected with PSaV Cowden strain (MOI of 1) for 36 h at 37 °C. Virus titers were determined at 6 days post-infection using the TCID_50_ assay as previously described [34], and TCID_50_/mL was calculated using the method of Reed and Muench [48].

### Statistical analyses and software

Statistical analyses were performed on triplicate experiments using GraphPad Prism 5 software version 5.03 (GraphPad Software, Inc., La Jolla, CA, USA) and a one-way ANOVA test. A *P* value of less than 0.05 was considered statistically significant. Figures were generated using Adobe Photoshop CS3 (Adobe Systems, San Jose, CA, USA) and the aforementioned Prism 5 version 5.03 software.

## Results

### PSaV infection requires clathrin, dynamin, and cholesterol

**Figure 1:**
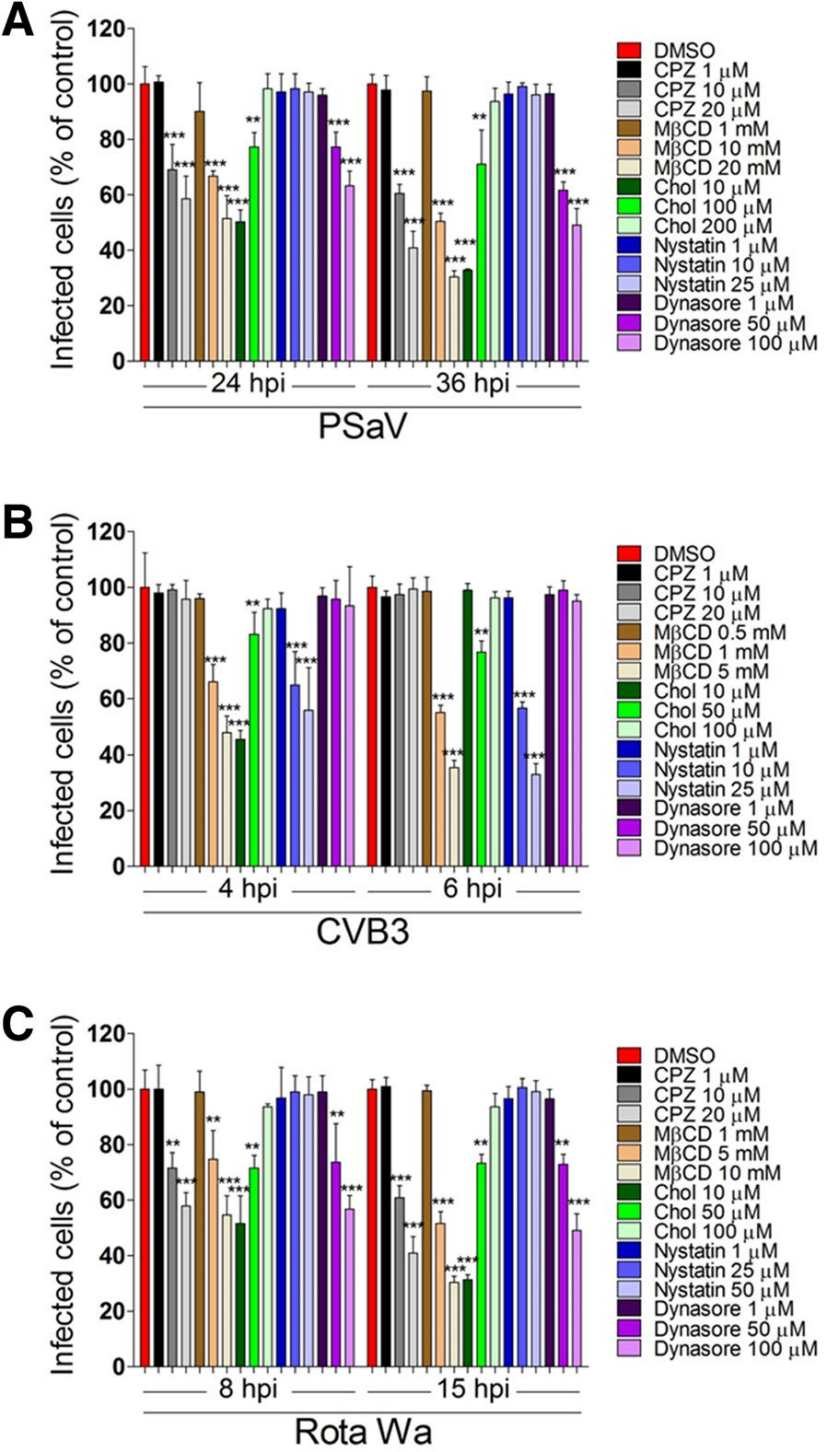
**Chemical inhibitors against clathrin-, dynamin-, and cholesterol-mediated endocytosis reduce PSaV infection. A**–**C** Confluent monolayers of LLC-PK, Caco-2, and MA104 cells were treated with DMSO vehicle or different concentrations of chlorpromazine (CPZ), MβCD, nystatin, or dynasore prior to infection with PSaV Cowden (**A**), Coxsackievirus B3 (CVB3) Nancy (**B**), or human rotavirus Wa (**C**) strains. Soluble cholesterol was added to the medium to examine the effect of cholesterol replenishment following MβCD-mediated depletion and then cells were infected with PSaV Cowden, CVB3 Nancy, or human rotavirus Wa strains. Infected cells were counted after staining with antibodies specific for each virus, and nuclei were counted after staining with DAPI. For each virus, results are shown as the percentage of infected cells, and normalized to the result obtained with control DMSO-treated cells. Data for panels **A**–**C** are presented as mean ± standard deviation of the mean from three independent experiments. Differences were evaluated using one-way ANOVA. *P < 0.05; **P < 0.001; ***P < 0.0001.

**Figure 2:**
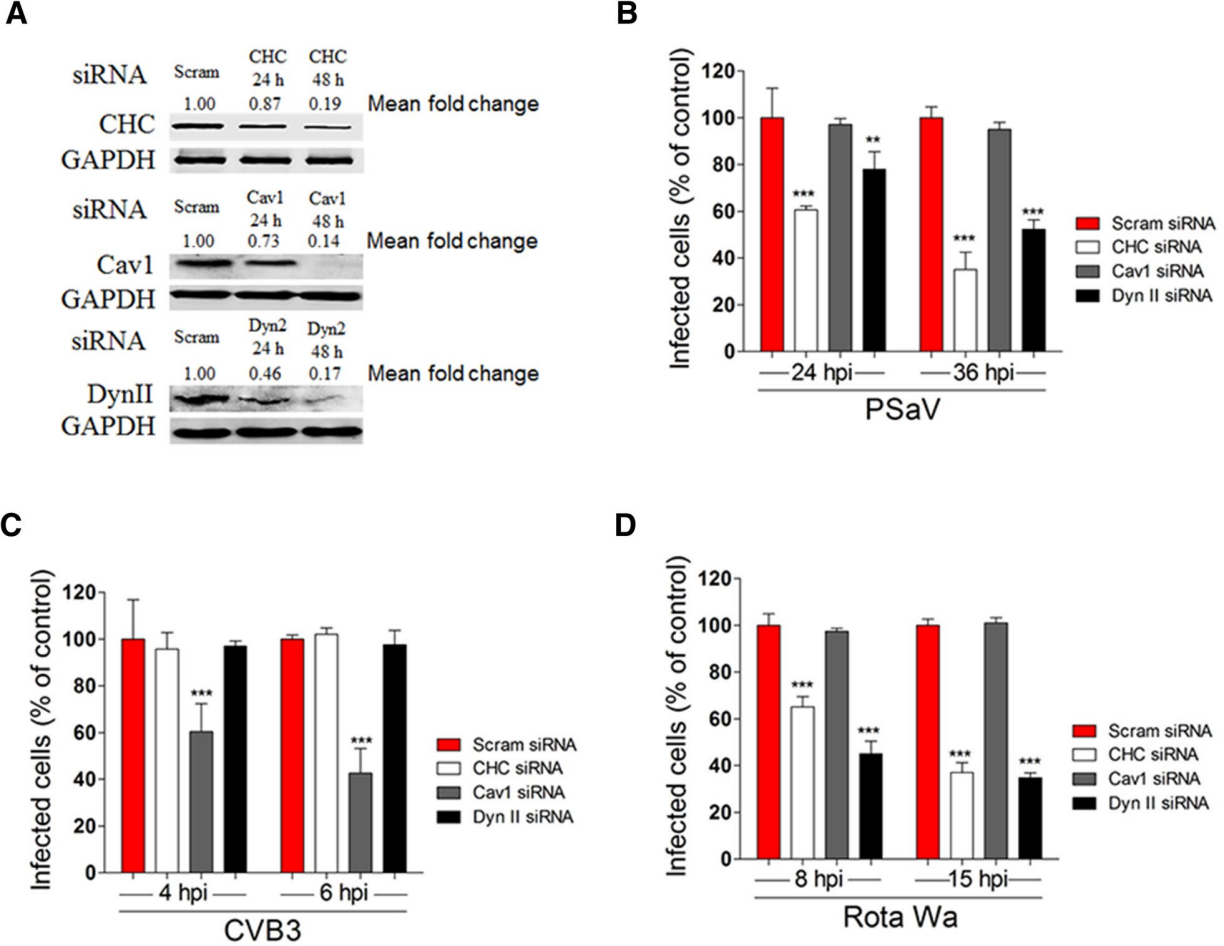
**Small interfering RNAs (siRNAs) against clathrin- and dynamin-mediated endocytosis reduce PSaV infection. A** LLC-PK cells transfected with scrambled siRNA (Scram) or siRNAs against clathrin heavy chain (CHC), dynamin II, or caveolin-1 were harvested at 24 and 48 h post-transfection. The down-regulation of each protein by siRNA knock-down was evaluated by western blotting analysis using antibodies specific for each protein. The intensity of each target protein relative to GAPDH was determined by densitometric analysis and is indicated above each lane. The cells transfected with each siRNA and then incubated were infected with PSaV Cowden (**B**), CVB3 Nancy (**C**), or rotavirus Wa (**D**) strains. Infected cells were counted after staining with antibodies specific for each virus, and nuclei were counted after staining with DAPI. For each virus, results are shown as the percentage of infected cells, and normalized to the results obtained in the scrambled siRNA-transfected cells. Data for panels **B**–**D** are presented as mean ± standard deviation of the mean from three independent experiments. Differences were evaluated using one-way ANOVA. *P < 0.05; **P < 0.001; ***P < 0.0001.

**Figure 3:**
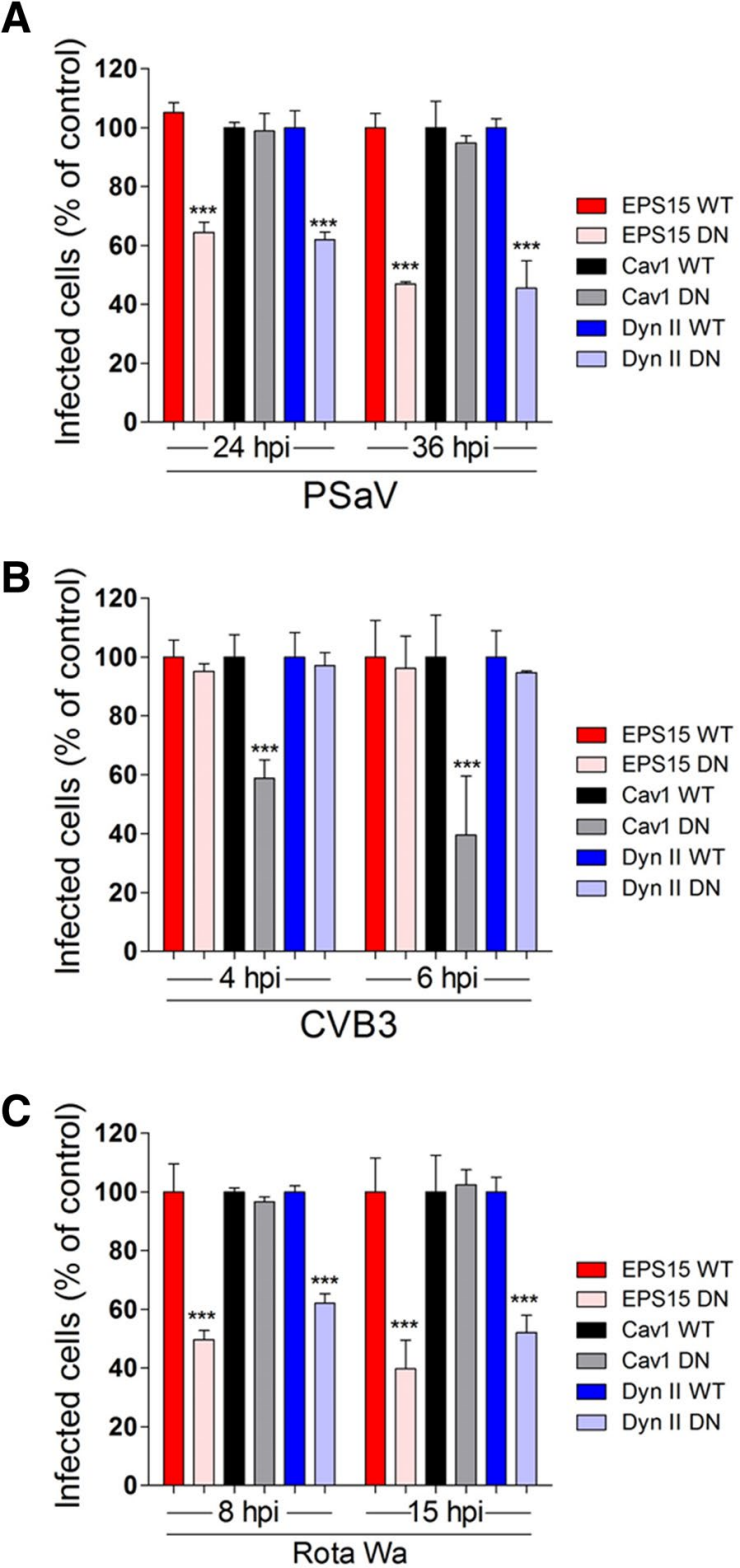
**Dominant negative (DN) mutants against clathrin- and dynamin-mediated endocytosis reduce PSaV infection.** The cells transfected with GFP-tagged wild type (WT) or dominant negative (DN) Eps15, caveolin-1, or dynamin II were incubated with PSaV Cowden (**A**), CVB3 Nancy (**B**), or rotavirus Wa (**C**) strains. Infected GFP-expressing cells were counted after being stained with antibodies specific for each virus. Data for panels **A**–**C** are presented as mean ± standard deviation of the mean from three independent experiments. Differences were evaluated using one-way ANOVA. *P < 0.05; **P < 0.001; ***P < 0.0001.

To examine the cellular factors required for PSaV entry into cells, we used pharmacological inhibitors after confirming their cytotoxicity (Additional files 2, 3, 4), siRNAs against clathrin heavy chain, dynamin II, and caveolin-1, and dominant negative (DN) mutants targeting clathrin- and non-clathrin-mediated endocytosis. PSaV infection was significantly inhibited by chlorpromazine (Figure 1), an inhibitor of clathrin-mediated endocytosis [49], by siRNA depletion of clathrin (Figure 2), and by a DN mutant of the clathrin adaptor protein Eps15 (Figure 3). PSaV infection was also markedly inhibited by dynasore (Figure 1), a pharmacological inhibitor of dynamin GTPase activity [50], by siRNA depletion of dynamin II (Figure 2), and by a DN mutant of dynamin II (Figure 3). In addition, methyl-beta cyclodextrin (MβCD), a pharmacological inhibitor of cholesterol-mediated endocytosis [30], also had a marked inhibitory effect on PSaV infection, but this inhibitory effect was restored by addition of soluble cholesterol (Figure 1). In contrast, treatment with nystatin, which disrupts caveolae-mediated endocytosis, depletion of caveolin-1 by siRNA, or transfection of a DN mutant for caveolin-1 had no effect on PSaV infection (Figures 1, 2, 3). However, infection of Caco-2 cells by the control CVB3 Nancy strain, which is cholesterol- and caveola/lipid raft-dependent [30] was inhibited by MβCD and nystatin (Figure 1), by depletion of caveolin-1 using siRNAs (Figure 2), and by the use of a DN mutant of caveolin-1 (Figure 3). Similarly, infection of cells with the human rotavirus Wa strain, which uses a clathrin-, cholesterol-, and dynamin-dependent entry process [51, 52], was significantly inhibited by chlorpromazine, MβCD, and dynasore (Figure 1), by siRNA depletion of clathrin or dynamin (Figure 2), as well as by DN mutants of the clathrin adaptor protein Eps15 and dynamin II (Figure 3). These results indicate that infection of permissive LLC-PK cells by the PSaV Cowden strain requires clathrin, cholesterol, and dynamin II.

### Use of light-sensitive PSaV to assess PSaV entry

**Figure 4:**
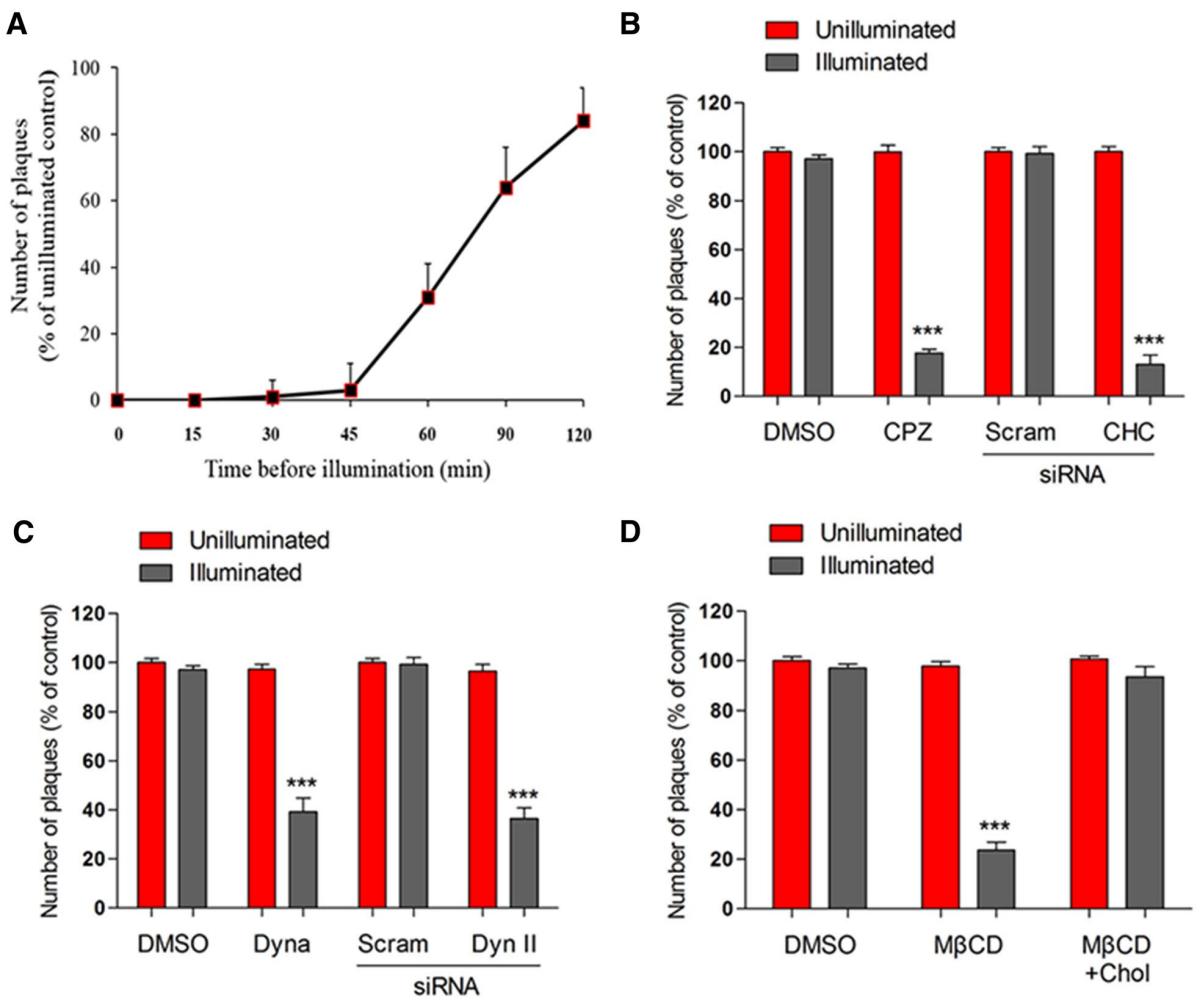
**PSaV entry and RNA release depend on clathrin-, dynamin-, and cholesterol-mediated endocytosis. A** LLC-PK cells grown in 6-well plates were incubated with neutral red (NR)-labeled PSaV Cowden strain for the indicated times and then exposed or not to UV light. The supernatants of each cell lysate were inoculated into fresh LLC-PK monolayers in 6-well plates, overlaid with agar, and incubated for 4 days. Results are shown as percentages to the number of plaques in the unilluminated control. The NR infectious center assay was performed in cells pretreated with DMSO vehicle or 20 μM chlorpromazine (CPZ), or transfected with scrambled siRNA or CHC siRNA (**B**); pretreated with DMSO vehicle or 100 μM dynasore (Dyna), or transfected with scrambled siRNA or dynamin II (Dyn II) siRNA (**C**); or pretreated with DMSO vehicle, 20 mM MβCD for 1 h, or MβCD followed by incubation with 200 μM soluble cholesterol for 30 min (**D**). The cells were then incubated with NR-labeled PSaV for 120 min. The number of plaques was normalized to those obtained with unilluminated controls exposed to the above conditions. Data for panels **A**–**D** are presented as mean ± standard deviation of the mean from three independent experiments. Differences were evaluated using one**-**way ANOVA. *P < 0.05; **P < 0.001; ***P < 0.0001.

To gain a better understating of the kinetics of PSaV entry and uncoating into permissive LLC-PK cells, we used a NR infectious center assay as previously described [31, 47]. The RNA-binding NR dye is passively incorporated into the viral RNA during viral propagation, resulting in light-sensitive NR-containing virions. Upon UV illumination, the dye is activated, damaging the viral RNA, and inactivating the virus infectivity [31, 47]. However, after virus entry, uncoating, and release of the viral genome into the cytoplasm, the NR dissociates from the viral RNA genomes, which thereby become light insensitive [31, 47]. NR-labeled MNV has previously been used to study virus entry, highlighting the utility of NR in the study of calicivirus entry [26]. As shown in Figure 4A, NR-labeled PSaV Cowden strain was sensitive to light exposure from 45 min post-infection (mpi), but the majority of the strain became insensitive to light from 90 mpi. This agrees with our previous finding that uncoating and genome release of PSaV Cowden strain take place within 90 mpi [29, 30].

Using the NR-containing PSaV Cowden strain, we further examined the specific role of clathrin, dynamin, and cholesterol in PSaV uncoating and genome release. Treatment with DMSO vehicle alone prior to infection of PSaV Cowden strain had no inhibitory effect on PSaV-induced infectious center formation at 120 mpi regardless of UV illumination (Figures 4B–D). However, light exposure at 120 mpi significantly reduced PSaV-induced infectious center formation after pretreatment with chlorpromazine, dynasore, and MβCD, and after siRNA depletion of clathrin and dynamin II in comparison with that of the equivalent unilluminated controls (Figures 4B–D). In addition, supplementation of cholesterol into the medium after MβCD treatment reinstated the infectious center formation by PSaV Cowden strain (Figure 4D). These results confirmed that entry of PSaV Cowden strain depends on clathrin, dynamin, and cholesterol.

### PSaV entry depends on clathrin, dynamin, and cholesterol

We next examined whether clathrin, dynamin, and cholesterol were also necessary for internalization of PSaV Cowden strain. LLC-PK cells were pretreated with vehicle or inhibitors, and the internalization of AF594-labeled PSaV Cowden strain was monitored as described elsewhere [31, 46]. In cells pretreated with either DMSO vehicle or nystatin, AF594-labeled PSaV particles were readily internalized in the cytoplasm (Additional file 5) and were evident around the nucleus (Additional file 5). However, pretreatment of the cells with chlorpromazine, dynasore, or MβCD trapped PSaV particles around the cell surface (Additional file 5). Replenishment of MβCD-treated cells with soluble cholesterol restored the internalization of AF594-labeled PSaV particles, leading to their internalization and accumulation of virus particles in the perinuclear area (Additional file 5). These findings, together with the data presented above, indicate that entry and infection of PSaV Cowden strain require the internalization of virions through both clathrin- and cholesterol-mediated endocytosis, in a process that also requires dynamin II.

### Transit of PSaV from EEs to LEs

We previously demonstrated that LEs are involved in PSaV trafficking [29, 30]. However, the precise mechanism by which PSaV Cowden strain reaches LEs is unclear. We examined the localization of AF594-labeled PSaV Cowden strain during the entry phase by co-staining cells with markers for EEs (EEA1) and LEs (LAMP2). Results showed that AF594-labeled PSaV Cowden strain was found maximally associated with the EE marker EEA1 at 30 mpi, after which the colocalization decreased, as did the signal for AF594-labeled PSaV Cowden strain (Additional file 6). The colocalization of PSaV Cowden strain with the LE marker LAMP2 was maximal at 60 mpi, after which the signal for AF594-labeled virus particles decreased (Additional file 6B), indicating that uncoating and genome release of PSaV Cowden strain had most likely already occurred at 90 mpi. These results further support our previous findings [29, 30] and agree with the results obtained using the NR infectious center assays (Figure 4A), suggesting that PSaV Cowden strain travels from EEs to LEs, and that its viral genome release occurs by 90 mpi.

### Role of Rab proteins in PSaV entry and infection

**Figure 5:**
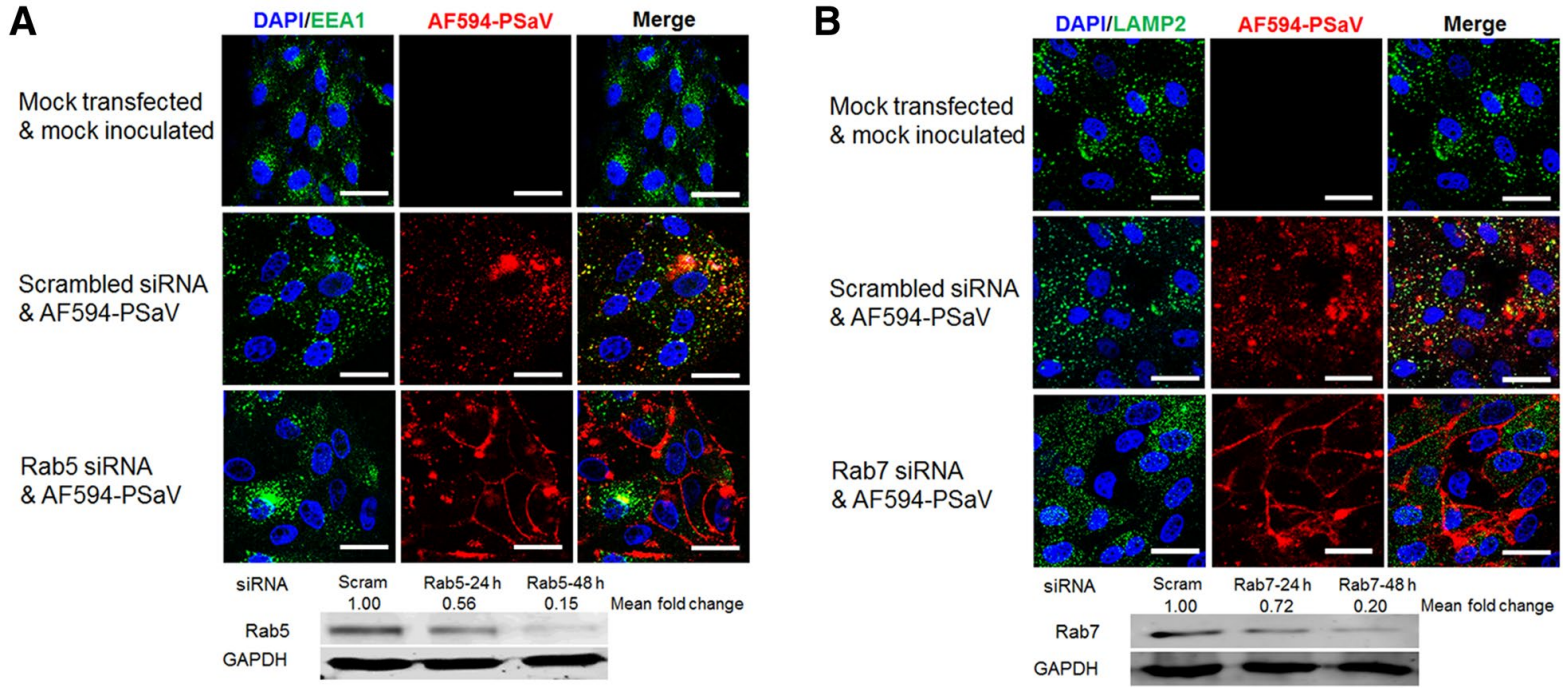
**PSaV entry depends on Rab5 and Rab7. A**, **B** LLC-PK cells were transfected with scrambled siRNA or siRNAs against Rab5 (**A**) or Rab7 (**B**). Next, cells were exposed to AF594-labeled PSaV particles (approximately 415 particles per cell) for EEA1 colocalization or for LAMP2 colocalization. This experiment was done in triplicate and representative images are shown. The scale bars correspond to 10 μm. The bottom portion displays immunoblots to confirm silencing levels of Rab5 and Rab7 by transfection of each corresponding siRNA. The intensity of each target protein relative to GAPDH was determined by densitometric analysis and is indicated above each lane.

The Rab GTPases proteins are important regulators of the process of endocytosis including vesicle formation, vesicle movement, and membrane fusion [53]. Among these proteins, Rab5 regulates early endocytic events, while Rab7 is involved in the transition from EEs to LEs and the maturation of LEs [53]. To further define the entry process of PSaV Cowden strain, we examined whether Rab5 and Rab7 are required for virus entry and infection. As expected, AF594-labeled PSaV Cowden strain colocalized with EEA1 at 30 mpi (Figure 5A) and LAMP2 at 60 mpi (Figure 5B) in the perinuclear areas of scrambled siRNA-transfected LLC-PK cells. However, siRNA-mediated silencing of Rab5 or Rab7 trapped AF594-labeled virus particles in the periphery of the cytoplasm and abrogated the colocalization of virus particles with EEA1 or LAMP2, respectively (Figures 5A and B).

**Figure 6:**
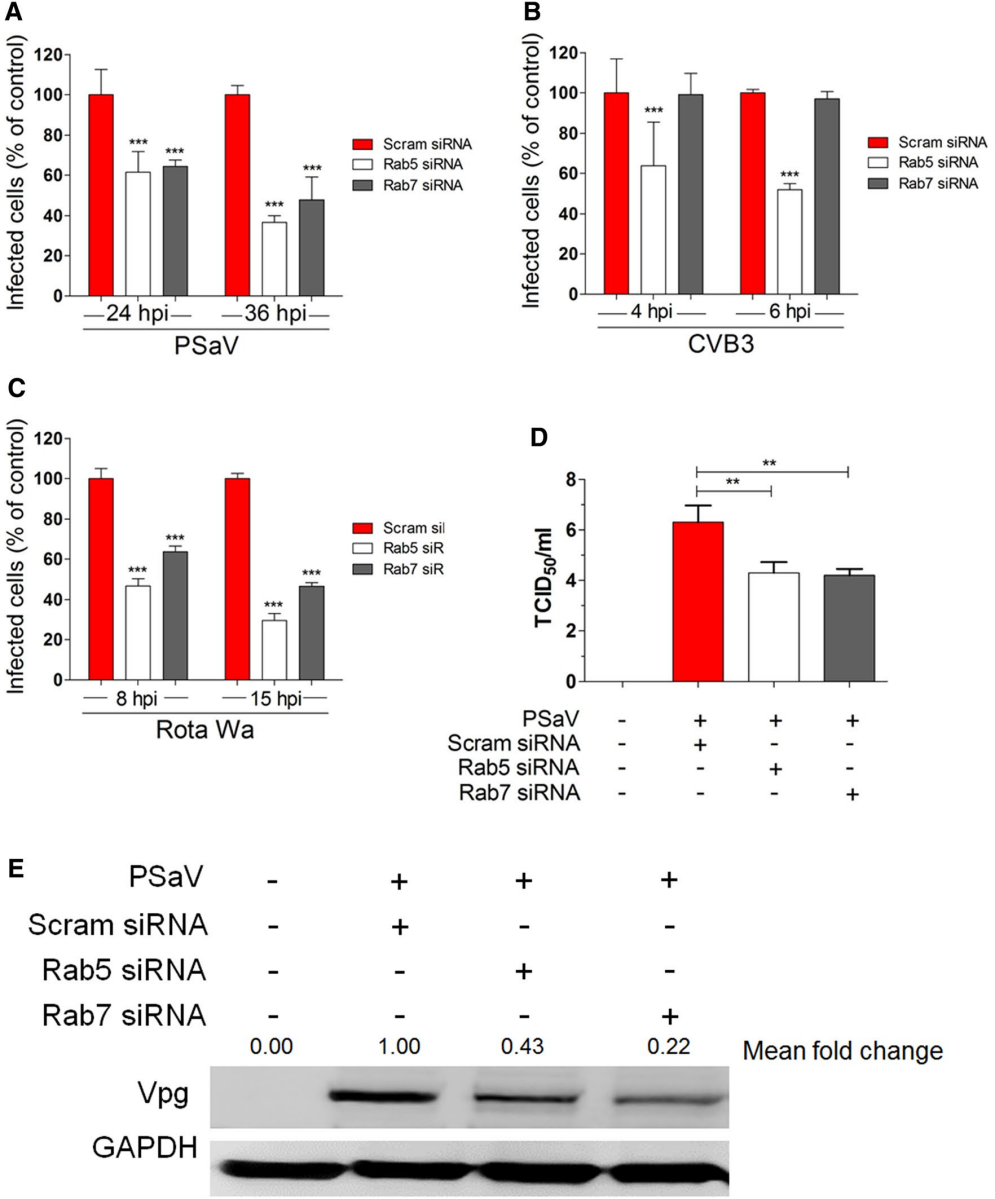
**PSaV infection depends on Rab5 and Rab7. A**–**D** LLC-PK, Caco-2, and MA104 cells were transfected with scrambled siRNA or siRNAs against Rab5 or Rab7, and then incubated with PSaV Cowden (**A**), CVB3 Nancy (**B**), or rotavirus Wa (**C**) strains, respectively. Infected cells were counted after staining with antibodies specific for each virus, and nuclei were counted after staining with DAPI. For each virus, results are shown as the percentage of infected cells, normalized to the results obtained in the scrambled siRNA-transfected cells. **D** The virus titer was determined by TCID_50_ using the cell lysates harvested from the cells in the above experimental conditions. Data for panels **A**–**D** are presented as mean ± standard deviation of the mean from three independent experiments. Differences were evaluated using the one-way ANOVA. *P < 0.05; **P < 0.001; ***P < 0.0001. **E** The level of PSaV VPg protein production in the above conditions was determined by western blotting analysis. GAPDH was used as a loading control. The intensity of PSaV VPg protein relative to GAPDH was determined by densitometric analysis and is indicated above its lane.

siRNA-mediated depletion of either Rab5 or Rab7 significantly reduced the number of virus-infected cells (Figure 6A), production of viral progeny (Figure 6D), and PSaV-VPg expression levels (Figure 6E) in comparison with scrambled siRNA-transfected cells. As shown in Figure 6B, infectivity of the EE-dependent CVB3 Nancy strain was significantly decreased only by Rab5 depletion [31, 54], whereas entry of the LE-dependent rotavirus Wa strain was significantly reduced by siRNA-mediated depletion of both Rab5 and Rab7 (Figure 6C) [55]. Taken together, these results indicate that PSaV Cowden strain travels from EEs to LEs before uncoating occurs.

### Endosomal acidification, actin, and microtubules are required for PSaV entry and infection

**Figure 7:**
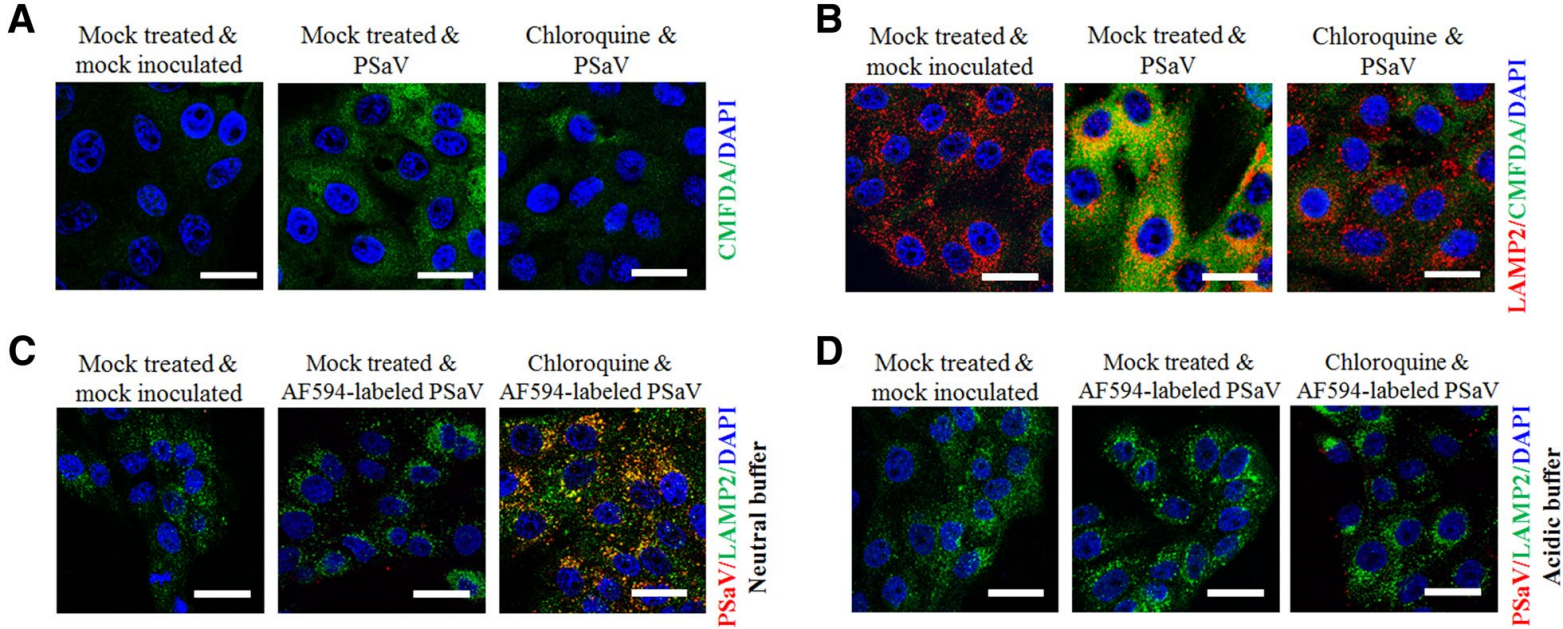
**PSaV entry is pH-dependent and involves actin and microtubules. A**, **B** Mock- or chloroquine-treated LLC-PK cells were inoculated with the PSaV Cowden strain and then incubated with CMFDA to visualize acidification of intracellular compartments, and incubated for further 30 min in maintenance media (**A**) or incubated with CMFDA followed by LAMP2 antibody to check colocalization by confocal microscopy (**B**). **C**, **D** After pretreatment of LLC-PK cells with chloroquine and infection with AF594-labeled PSaV particles (approximately 415 particles per cell), cells were incubated with neutral (pH 7.2) (**C**) or acidic (pH 5.2) buffers (**D**). The cells were prepared for confocal microscopy to check colocalization of AF594-labeled PSaV particles with LAMP2. All experiments were performed in triplicate and panels **A**–**D** show representative sets of results. Scale bars for panels **A**–**D** correspond to 10 μm.

We have recently shown that PSaV Cowden strain requires acidification of LEs for efficient entry and infection [29, 30]. To explore this in more detail, we examined endosomal acidification after infection of PSaV Cowden strain using the green CMFDA pH probe. As shown in Figure 7A, the intensity of the CMFDA-positive fluorescent signal markedly increased in virus-infected cells, and significantly decreased after pretreatment of cells with chloroquine, an inhibitor of endosomal acidification. Colocalization of the CMFDA-positive structures with LAMP2 was not observed in the control cells but appeared in virus-infected cells (Figure 7B). However, pretreatment of cells with chloroquine markedly decreased the intensity of CMFDA staining, which resulted in reduced colocalization with LAMP2 (Figure 7B). To confirm the above results, we next investigated whether replacement of the culture medium with an acidic buffer in chloroquine-pretreated cells could restore virus release from LEs. As expected, replacement of the culture medium with a neutral buffer still trapped AF594-labeled virus particles in the LEs of chloroquine-pretreated cells even after incubation for 2 h (Figure 7C). Interestingly, acidic replenishment of the culture medium resulted in the disappearance of viral particles from the LEs in chloroquine-pretreated cells (Figure 7D). Furthermore, PSaV-infection was reduced in chloroquine-pretreated cells in a dose-dependent manner (Additional file 7). These data further confirm that entry and infection of PSaV Cowden strain depends on acidification of LEs [29, 30].

We next examined the role of the actin cytoskeleton and microtubules in the vesicular trafficking of PSaV. Treatment with either the actin-polymerizing inhibitor, cytochalasin D, or the microtubule disrupter, nocodazole, reduced the number of PSaV-infected cells in a dose-dependent manner (Additional file 7). These results suggest that actin remodeling and microtubules are required for PSaV entry and infection. In contrast, pretreatment of cells with amiloride, an inhibitor of macropinocytosis, had no inhibitory effect on virus infection, indicating that PSaV Cowden strain does not enter the cells through macropinocytosis (Additional file 7).

To confirm that PSaV Cowden strain does not use caveolin-mediated endocytosis or macropinocytosis as a minor route of entry, we pretreated LLC-PK cells with a mixture of inhibitors. Chlorpromazine was used alone or in combination with nystatin or amiloride. In addition, MβCD was used alone or with nystatin or amiloride. After the pretreatments, the cells were infected with PSaV Cowden strain. Results showed that no significant decrease in PSaV entry was observed in the combined treatments in comparison with the sole use of chlorpromazine or MβCD (Additional file 8), suggesting that caveolin-mediated endocytosis and macropinocytosis did not play a minor role in PSaV infection in LLC-PK cells.

## Discussion

The lack of an efficient cell culture system of human SaVs has hampered the study of the SaV life cycle. PSaV Cowden strain remains the only cultivable member of the genus *Sapovirus* and thus has been widely used as a model strain [23, 33, 46]. Although receptor-mediated entry of viruses into host cells represents the first step of virus infection, the precise mechanisms utilized by the members of the *Sapovirus* genus to enter permissive cells have not been defined. Here, we demonstrate that PSaV Cowden strain is internalized into LLC-PK cells via clathrin-, cholesterol-, and dynamin-mediated endocytosis. The entry of PSaV Cowden strain has apparently similar features to those of other members of the *Caliciviridae*. For instance, the FCV F9 strain in the genus *Vesivirus* utilizes clathrin-mediated endocytosis [24], whereas entry of MNV-1 from the *Norovirus* genus occurs via a cholesterol- and dynamin-dependent pathway [26].

Dynamin is a large GTPase that is best known for its role in plasma membrane fission and pinching off of endocytic vesicles from the cellular plasma membrane during clathrin-dependent endocytosis [2, 26]. Many viruses, such as vesicular stomatitis virus [56] and classical swine fever virus [57], enter the cells through a clathrin-dependent pathway in cooperation with dynamin II. Within the *Caliciviridae* family, FCV uses clathrin-mediated endocytosis but the role of dynamin is unclear [24]. Our results suggest that dynamin mediates the fission and detachment of clathrin-coated pits for entry of PSaV Cowden strain.

Dynamin is also involved in plasma membrane fission and pinching off of endocytic vesicles in caveolin-1- or cholesterol-dependent endocytosis from the cellular plasma membrane [2, 26]. Cholesterol-/dynamin II-dependent endocytosis plays a role in the entry of several viruses, such as MNV-1 [26], CVB3 [58], feline infectious peritonitis virus (FIPV) [59], and the simian rotavirus RRV strain [52]. In addition, a study using the Norwalk replicon system demonstrated the importance of cholesterol in the replication of HuNoV [60]. In the present study, inhibition of the entry and infection of PSaV Cowden strain by the cholesterol-sequestering drug MβCD was restored by the addition of soluble cholesterol. These data indicate that, like MNV-1 [26], PSaV Cowden strain also utilizes cholesterol-/dynamin II-dependent endocytosis. In contrast, by depleting caveolin-1 using nystatin, siRNA, or a DN mutant for caveolin-dependent endocytosis, we demonstrated that entry of PSaV Cowden strain does not require caveolin-/dynamin II-dependent endocytosis. We also investigated the role of alternative or minor routes of PSaV entry by co-treatment with inhibitors. However, no combination of inhibitors produced a significant decrease in the rate of infection of PSaV Cowden strain over chlorpromazine or MβCD alone. Since cholesterol-dependent endocytosis is still poorly defined [2], the reason why the two cholesterol-sequestering drugs (MβCD and nystatin) have a different effect on the cholesterol- and dynamin-dependent entry of PSaV Cowden strain remains unknown. Nevertheless, it has been reported that the sensitivity of different cholesterol-dependent viruses to cholesterol-sequestering drugs can be different. For example, FIPV is sensitive to nystatin but not to MβCD [59], whereas, similar to what we observed with the human rotavirus Wa strain in this study, the simian rotavirus RRV strain is sensitive to MβCD but not to nystatin [51, 52]. In addition, CVB3 is sensitive to both MβCD and nystatin [31].

Recent reports have shown that PSaV Cowden strain requires bile acids, acidification, and cathepsin L activity in Rab7-positive LEs for efficient trafficking and uncoating [29, 30]. However, the detailed mechanism used by PSaV Cowden strain to travel from EEs to LEs has been unclear. In the present study, PSaV Cowden strain maximally colocalized with the EE marker EEA1 at 30 mpi, and with the LE marker LAMP2 at 60 mpi, suggesting that PSaV Cowden strain travels from EEs to LEs. Moreover, depletion of either Rab5 or Rab7 by corresponding specific siRNA trapped PSaV Cowden strain in the periphery of the cytoplasm. The data confirm that, similar to FCV trafficking [24], entry and infection of PSaV Cowden strain require endosomal trafficking from EEs to LEs [29, 30].

Pretreatment of cells with cytochalasin D significantly reduced the infectivity of PSaV Cowden strain, suggesting that rearrangement of actin filaments plays an important role in virus internalization. Actin has been reported to have different functions in the calicivirus life cycle for FCV and MNV [24–26]. Cytochalasin D can inhibit the later stages of the FCV life cycle, with no inhibitory effect during FCV entry [24]. In contrast, infection of MNV-1 is somewhat increased by treatment with cytochalasin D [25, 26]. Further studies are required to examine whether post-treatment with cytochalasin D inhibits the replication of PSaV Cowden strain in virus-infected cells or in cells transfected with the viral RNA genome. The present finding that infectivity of PSaV Cowden strain diminished in LLC-PK cells pretreated with the microtubule-disrupting nocodazole suggests a similar mechanism to the one used by MNV-containing endosomes to move deeper into the cytoplasm using microtubules as an intracellular highway [25].

Endosomal acidification plays a crucial role during uncoating of most viruses [58, 59]. Interestingly, the dependency of endosomal acidification varies with different caliciviruses. For example, endosomal acidification is required by FCV [24] but not by MNV [25, 26, 28]. However, the dependency of endosomal acidification for MNV infection is controversial. Two recent reports described that the inhibition of endosomal acidification and of cathepsin L, which requires acidic pH for optimal activity, significantly reduced the replication of MNV, FCV, and PSaV [29, 30]. In the present study, inhibition of endosomal acidification by chloroquine treatment significantly reduced the fluorescence intensity of the CMFDA pH probe in parallel with loss of colocalization with LAMP2 in PSaV-infected cells, suggesting that late endosomal acidification is necessary for PSaV uncoating. Furthermore, replenishment of the medium of chloroquine-treated cells with an acidic buffer induced the escape of PSaV Cowden strain from LEs and restored the infectivity of PSaV Cowden strain, confirming that uncoating of PSaV Cowden strain requires acidification of LEs. Consistent with our previous reports [29, 30] and similar to what has been observed in FCV [24], the findings presented here indicate that an endosomal low pH is indispensable for the uncoating of PSaV Cowden strain.

In conclusion, we provide evidence that PSaV Cowden strain enters permissive LLC-PK cells via clathrin- and cholesterol-dependent endocytic pathways that require dynamin II for vesicle fission and detachment, as well as actin rearrangements for vesicle internalization. In addition, PSaV Cowden strain requires Rab5 and Rab7 GTPases to deliver viral particles from EEs to LEs along the microtubules and acidification of LEs for uncoating and genome release of PSaV Cowden strain into the cytoplasm. These findings provide new insights into the biology of these highly prevalent RNA viruses.

## Additional files

**Additional file 1.** **Sequences of siRNAs against target molecules and scrambled siRNA used in this study.**

**Additional file 2.** **Determination of chemical-mediated cytotoxicity in LLC-PK cells by MTT assay.** (A–I) LLC-PK cells grown in 96-well plates were incubated with various concentrations of the indicated chemicals in triplicate for 24 h at 37 °C. Afterward, the chemical-containing media was thoroughly removed and replaced with 200 μL of MTT solution for 4 h at 37 °C. Each well was incubated with 100 μL of DMSO for 10 min at room temperature. Cell viability was measured using an ELISA reader at an OD value of 570 nm. The arrows indicate the concentrations used in this study.

**Additional file 3.** **Determination of chemical-mediated cytotoxicity in Caco-2 cells by MTT assay.** (A–E) Caco-2 cells grown in 96-well plates were incubated with various concentrations of the indicated chemicals in triplicate for 24 h at 37 °C. Afterward, the chemical-containing media was thoroughly removed and replaced with 200 μL of MTT solution for 4 h at 37 °C. Each well was incubated with 100 μL of DMSO for 10 min at room temperature. Cell viability was measured using an ELISA reader at an OD value of 570 nm. The arrows indicate the concentrations used in this study.

**Additional file 4.** **Determination of chemical-mediated cytotoxicity in MA104 cells by MTT assay.** (A–E) MA104 cells grown in 96-well plates were incubated with various concentrations of the indicated chemicals in triplicate for 24 h at 37 °C. Afterward, the chemical-containing media was thoroughly removed and replaced with 200 μL of MTT solution for 4 h at 37 °C. Each well was incubated with 100 μL of DMSO for 10 min at room temperature. Cell viability was measured using an ELISA reader at an OD value of 570 nm. The arrows indicate the concentrations used in this study.

**Additional file 5.** **PSaV entry depends on clathrin-, dynamin-, and cholesterol-mediated endocytosis.** (A and B) Confluent monolayers of LLC-PK pretreated with chemicals were exposed to AF594-labeled PSaV particles (approximately 415 particles per cell) for 30 min at 4 °C. To examine the effect of cholesterol replenishment following MβCD-mediated depletion, soluble cholesterol (MβCD + cholesterol group) was added to the medium and then cells were exposed to AF594-labeled PSaV particles. Afterward, unbound virus was washed off, and the cells were shifted to 37 °C for 30 min (A) or 60 min (B). Cells were then fixed, stained with AF488-labeled phalloidin for actin, and processed for confocal microscopy. All the experiments were done in triplicate and representative images are shown. The scale bars in each panel correspond to 10 μm.

**Additional file 6.** **Transport of PSaV particles to early and late endosomes.** LLC-PK cells were incubated with AF594-labeled PSaV particles (approximately 415 particles per cell) for the indicated time, fixed, permeabilized, and processed for the immunofluorescence assay to determine the colocalization of AF594-labeled PSaV particles with the early endosomal marker EEA1 (A) and the late endosomal marker LAMP2 (B). All experiments were performed in triplicate and representative images are shown. The scale bars in each panel correspond to 10 μm.

**Additional file 7.** **PSaV infection is pH-dependent and involves actin and microtubules.** LLC-PK cells were either mock-treated or chemical-treated and then infected with PSaV Cowden strain. The cells were then stained with an antibody against the PSaV VPg protein and the number of virus-positive cells was counted by confocal microscopy. Results are shown as the percentages to the number of positive cells in the DMSO vehicle-treated control. All experiments were performed in triplicate. Data are presented as mean ± standard deviation of the mean from three independent experiments. Differences were evaluated using the one-way ANOVA. *P < 0.05; **P < 0.001; ***P < 0.0001.

**Additional file 8.** **Caveolin-mediated endocytosis and macropinocytosis are not used as a minor route for PSaV entry.** Confluent monolayers of LLC-PK were treated with DMSO, chlorpromazine (CPZ) alone (-), CPZ and nystatin, CPZ and amiloride, MβCD alone (-), MβCD and nystatin, or MβCD and amiloride prior to infection with the PSaV Cowden strain. The cells were then stained with an antibody against the PSaV VPg protein and the number of virus-positive cells was counted by confocal microscopy. Results are shown as the percentage of infected cells normalized to the results obtained with control DMSO-treated cells. Data are presented as mean ± standard deviation of the mean from three independent experiments. Differences were evaluated using the one-way ANOVA. *P < 0.05; **P < 0.001; ***P < 0.0001.
