# Supplementary material for: Porcine sapovirus Cowden strain enters LLC-PK cells via clathrin- and cholesterol-dependent endocytosis with the requirement of dynamin II
Source: Vet Res. 2018 Sep 17;49:92. doi: 10.1186/s13567-018-0584-0 (PMC6142377; doi:10.1186/s13567-018-0584-0)
Supplement: Supplementary file 1 — Additional file 1. Sequences of siRNAs against target molecules and scrambled siRNA used in this study. [file 13567_2018_584_MOESM1_ESM.docx]

**Additional file 1 Sequences of siRNAs against each target gene and scrambled siRNA used in this study.**

| Target gene | Set | Sequence (5’ to 3’) | References |
| --- | --- | --- | --- |
| Clathrin heavy chain | A | Sense: CCUGCGGUCUGGAGUCAACTT  Antisense: GUUGACUCCAGACCGCAGGTT | [35-37] |
|  | B | Sense: UGGAUCUCUUUGAAUACGGTT  Antisense: CCGUAUUCAAAGAGAUCCATT |  |
| Caveolin 1 | A | Sense: AACCAGAAGGGACACACAG  Antisense: CUGUGUGUCCCUUCUGGUU | [37-39] |
| Dynamin II | A | Sense: CCAUCAUGCACCUCAUGAUtt  Antisense: AUCAUGAGGUGCAUGAUGGtt | [37] |
| Rab5 | A | Sense: GCAAGUCCUAACAUUGUAAtt  Antisense: UUACAAUGUUAGGACUUGCtt | [40] |
|  | B | Sense: CCAAAGAAUGAACCACAAAtt  Antisense: UUUGUGGUUCAUUCUUUGGtt |  |
|  | C | Sense: GUACCCGUAAUUUGUAACAtt  Antisense: UGUUACAAAUUACGGGUACtt |  |
| Rab7 | A | Sense: GGAAGACAUCACUCAUGAAtt  Antisense: UUCAUGAGUGAUGUCUUCCtt | [40] |
|  | B | Sense: CCAGUAUGUGAAUAAGAAAtt  Antisense: UUUCUUAUUCACAUACUGGtt |  |
|  | C | Sense: GCGUUCUGGUAUUUGAUGUtt  Antisense: ACAUCAAAUACCAGAACGCtt |  |
| Scrambled | A | r(UUCUCCGAACGUGUCACGU)d(TT) | [40] |
