# Supplementary figures and images for: Porcine sapovirus Cowden strain enters LLC-PK cells via clathrin- and cholesterol-dependent endocytosis with the requirement of dynamin II
Source: Vet Res. 2018 Sep 17;49:92. doi: 10.1186/s13567-018-0584-0 (PMC6142377; doi:10.1186/s13567-018-0584-0)

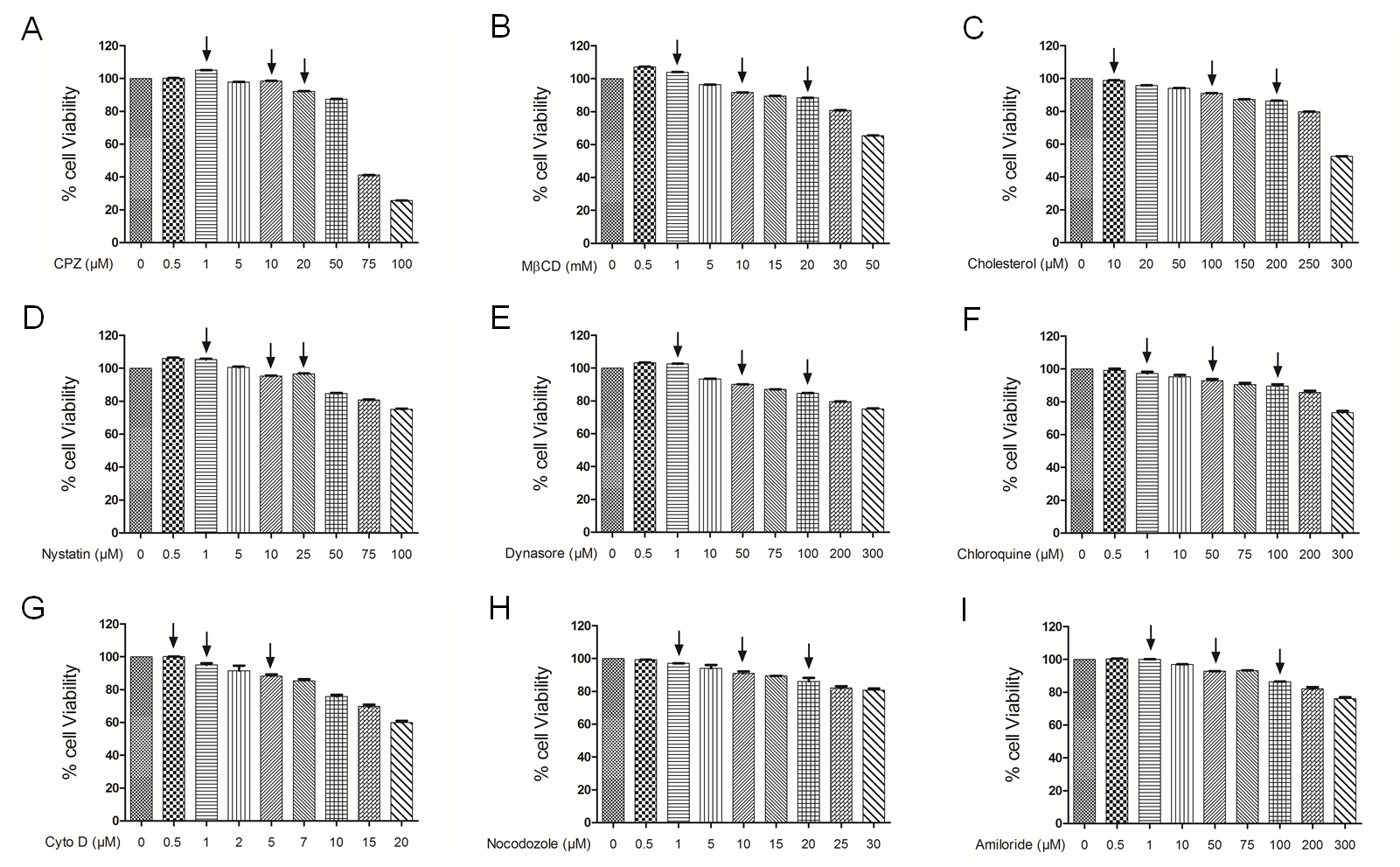

Supplement: Supplementary file 2 — Additional file 2. Determination of chemical-mediated cytotoxicity in LLC-PK cells by MTT assay. (A–I) LLC-PK cells grown in 96-well plates were incubated with various concentrations of the indicated chemicals in triplicate for 24 h at 37 °C. Afterward, the chemical-containing media was thoroughly removed and replaced with 200 μL of MTT solution for 4 h at 37 °C. Each well was incubated with 100 μL of DMSO for 10 min at room temperature. Cell viability was measured using an ELISA reader at an OD value of 570 nm. The arrows indicate the concentrations used in this study. [file 13567_2018_584_MOESM2_ESM.tif]

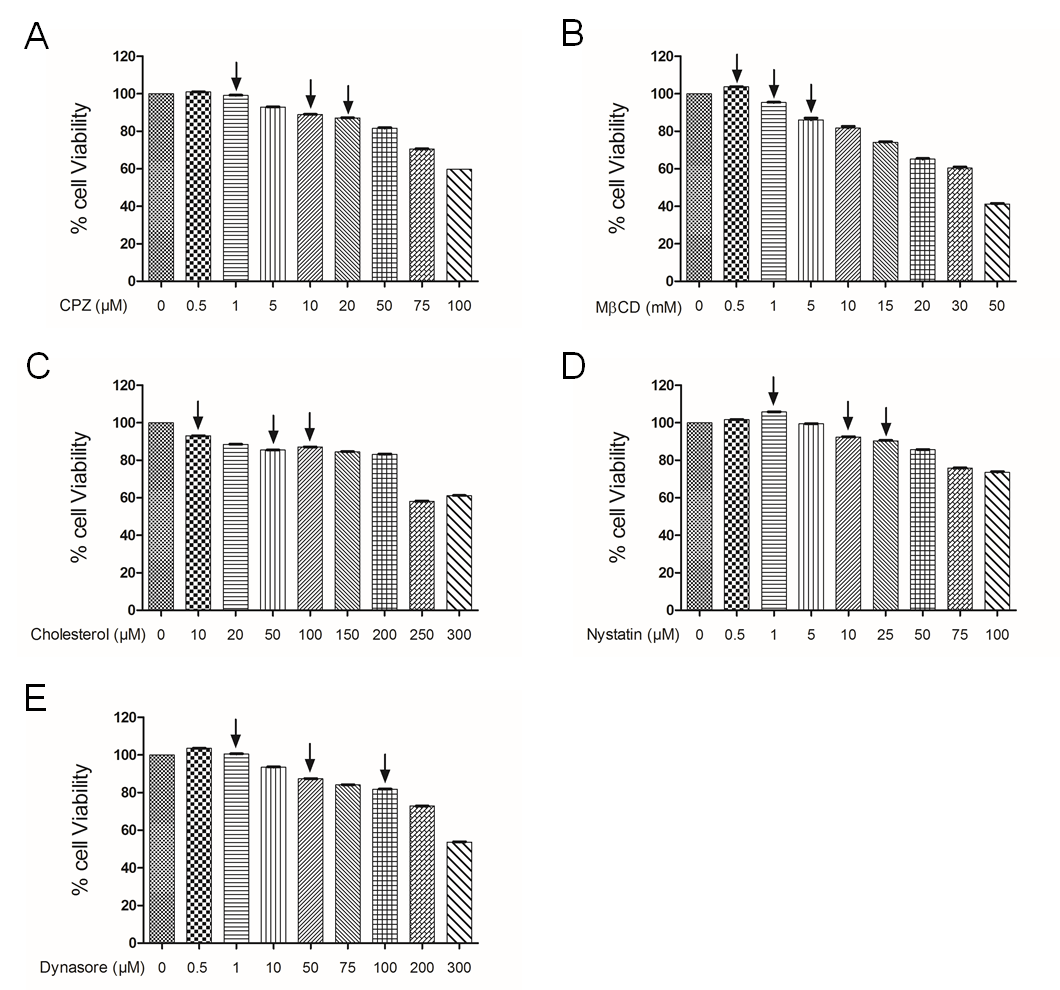

Supplement: Supplementary file 3 — Additional file 3. Determination of chemical-mediated cytotoxicity in Caco-2 cells by MTT assay. (A–E) Caco-2 cells grown in 96-well plates were incubated with various concentrations of the indicated chemicals in triplicate for 24 h at 37 °C. Afterward, the chemical-containing media was thoroughly removed and replaced with 200 μL of MTT solution for 4 h at 37 °C. Each well was incubated with 100 μL of DMSO for 10 min at room temperature. Cell viability was measured using an ELISA reader at an OD value of 570 nm. The arrows indicate the concentrations used in this study. [file 13567_2018_584_MOESM3_ESM.tif]

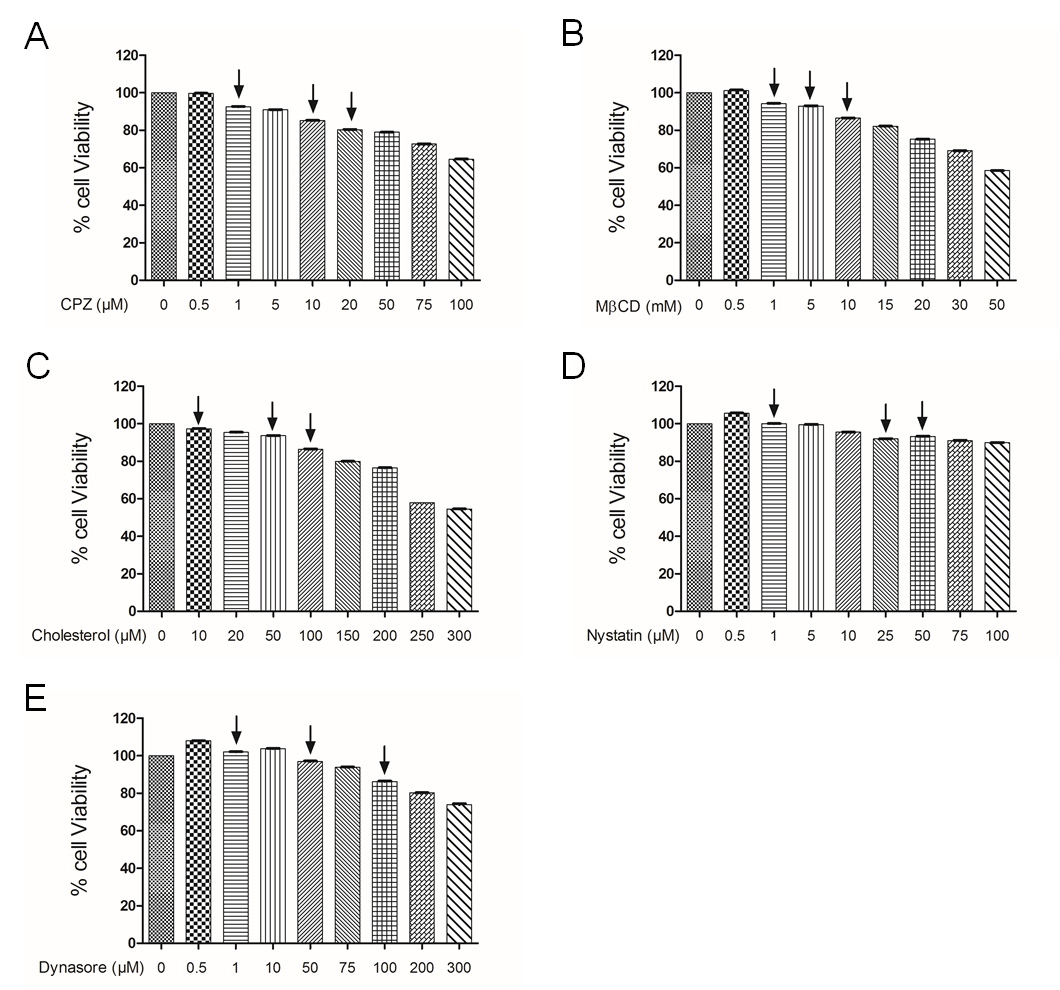

Supplement: Supplementary file 4 — Additional file 4. Determination of chemical-mediated cytotoxicity in MA104 cells by MTT assay. (A–E) MA104 cells grown in 96-well plates were incubated with various concentrations of the indicated chemicals in triplicate for 24 h at 37 °C. Afterward, the chemical-containing media was thoroughly removed and replaced with 200 μL of MTT solution for 4 h at 37 °C. Each well was incubated with 100 μL of DMSO for 10 min at room temperature. Cell viability was measured using an ELISA reader at an OD value of 570 nm. The arrows indicate the concentrations used in this study. [file 13567_2018_584_MOESM4_ESM.tif]

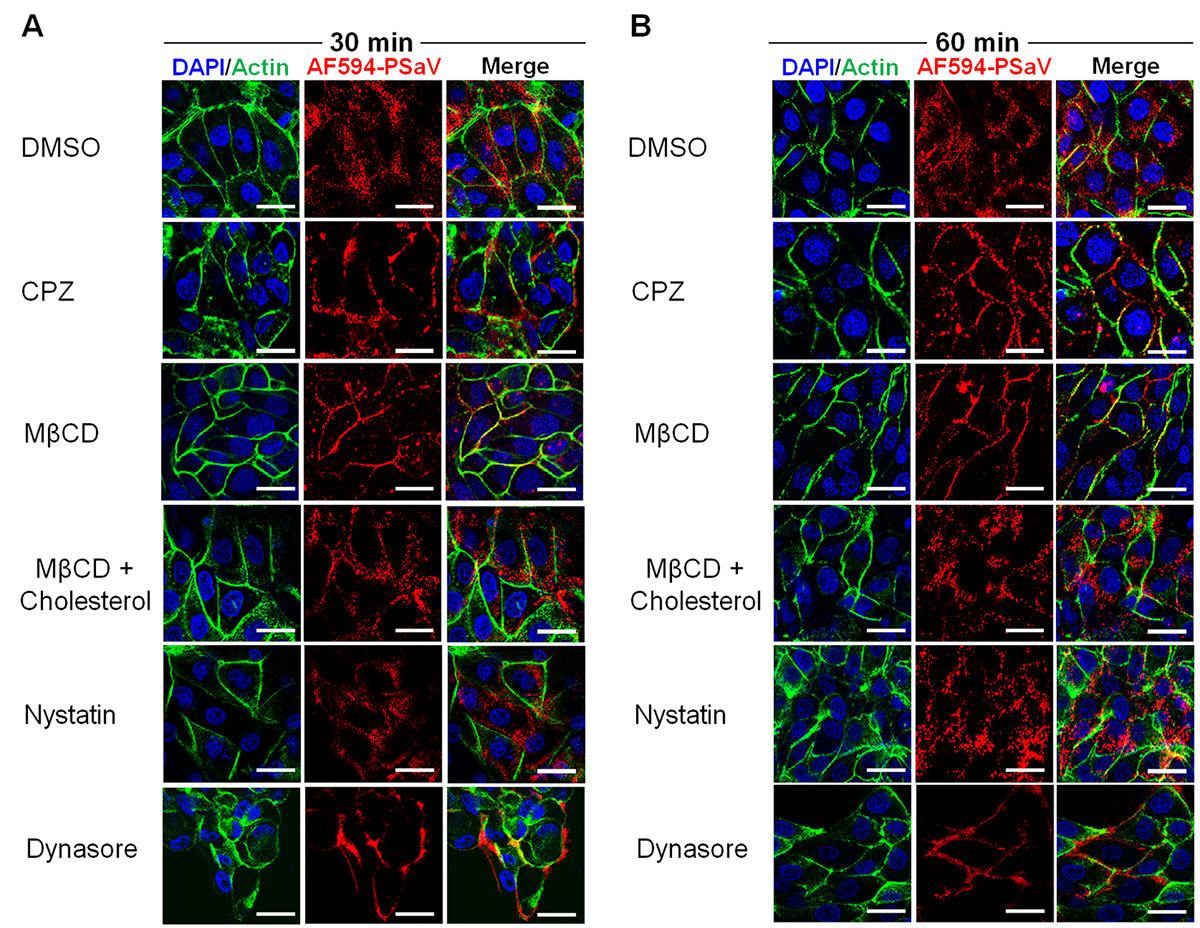

Supplement: Supplementary file 5 — Additional file 5. PSaV entry depends on clathrin-, dynamin-, and cholesterol-mediated endocytosis. (A and B) Confluent monolayers of LLC-PK pretreated with chemicals were exposed to AF594-labeled PSaV particles (approximately 415 particles per cell) for 30 min at 4 °C. To examine the effect of cholesterol replenishment following MβCD-mediated depletion, soluble cholesterol (MβCD + cholesterol group) was added to the medium and then cells were exposed to AF594-labeled PSaV particles. Afterward, unbound virus was washed off, and the cells were shifted to 37 °C for 30 min (A) or 60 min (B). Cells were then fixed, stained with AF488-labeled phalloidin for actin, and processed for confocal microscopy. All the experiments were done in triplicate and representative images are shown. The scale bars in each panel correspond to 10 μm. [file 13567_2018_584_MOESM5_ESM.tif]

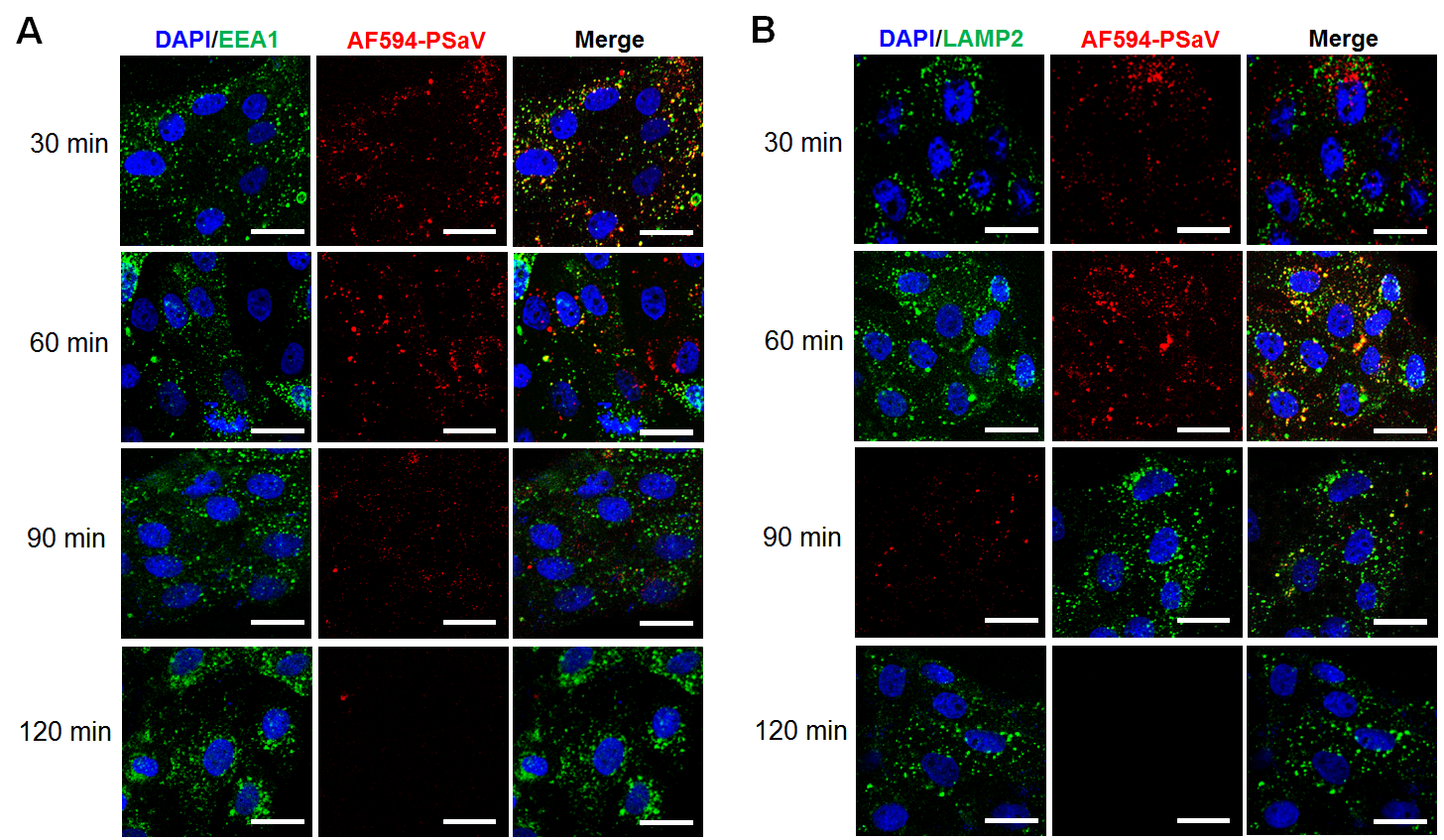

Supplement: Supplementary file 6 — Additional file 6. Transport of PSaV particles to early and late endosomes. LLC-PK cells were incubated with AF594-labeled PSaV particles (approximately 415 particles per cell) for the indicated time, fixed, permeabilized, and processed for the immunofluorescence assay to determine the colocalization of AF594-labeled PSaV particles with the early endosomal marker EEA1 (A) and the late endosomal marker LAMP2 (B). All experiments were performed in triplicate and representative images are shown. The scale bars in each panel correspond to 10 μm. [file 13567_2018_584_MOESM6_ESM.tif]

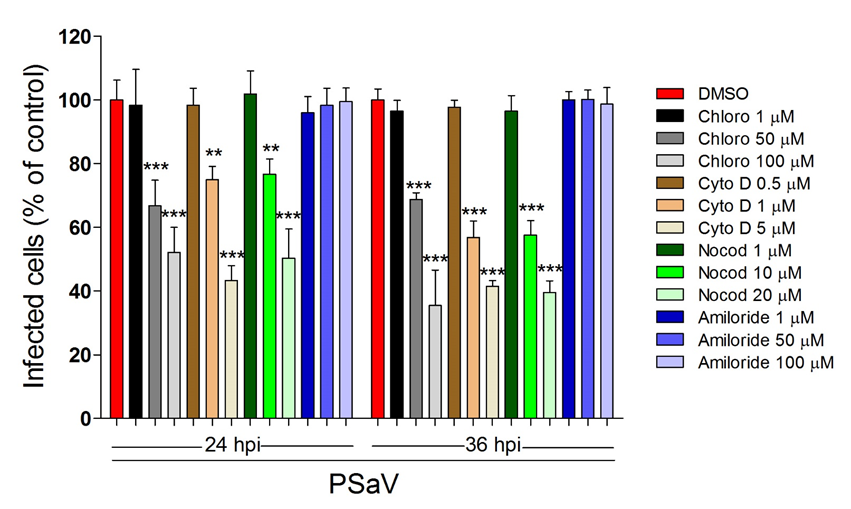

Supplement: Supplementary file 7 — Additional file 7. PSaV infection is pH-dependent and involves actin and microtubules. LLC-PK cells were either mock-treated or chemical-treated and then infected with PSaV Cowden strain. The cells were then stained with an antibody against the PSaV VPg protein and the number of virus-positive cells was counted by confocal microscopy. Results are shown as the percentages to the number of positive cells in the DMSO vehicle-treated control. All experiments were performed in triplicate. Data are presented as mean ± standard deviation of the mean from three independent experiments. Differences were evaluated using the one-way ANOVA. *P < 0.05; **P < 0.001; ***P < 0.0001. [file 13567_2018_584_MOESM7_ESM.tif]

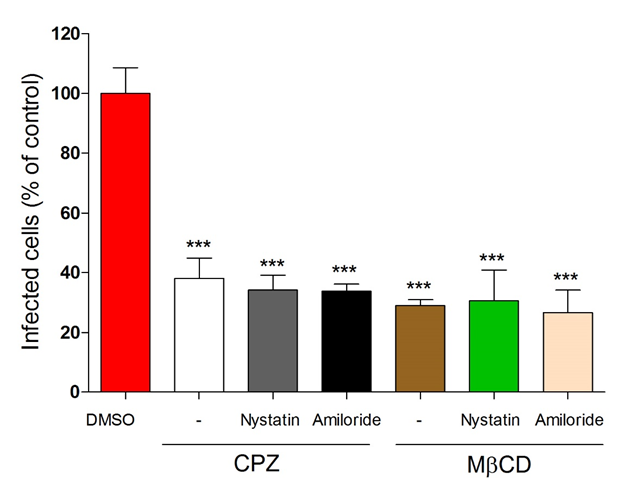

Supplement: Supplementary file 8 — Additional file 8. Caveolin-mediated endocytosis and macropinocytosis are not used as a minor route for PSaV entry. Confluent monolayers of LLC-PK were treated with DMSO, chlorpromazine (CPZ) alone (-), CPZ and nystatin, CPZ and amiloride, MβCD alone (-), MβCD and nystatin, or MβCD and amiloride prior to infection with the PSaV Cowden strain. The cells were then stained with an antibody against the PSaV VPg protein and the number of virus-positive cells was counted by confocal microscopy. Results are shown as the percentage of infected cells normalized to the results obtained with control DMSO-treated cells. Data are presented as mean ± standard deviation of the mean from three independent experiments. Differences were evaluated using the one-way ANOVA. *P < 0.05; **P < 0.001; ***P < 0.0001. [file 13567_2018_584_MOESM8_ESM.tif]
